# “Time-based” workplace smoking bans during working hours (including and excluding lunchtime) and combustible cigarette and heated tobacco product use: a cross-sectional analysis of the 2020 JASTIS study

**DOI:** 10.1016/j.pmedr.2022.101938

**Published:** 2022-07-29

**Authors:** Yuki Miyazaki, Takahiro Tabuchi

**Affiliations:** a Department of Psychiatry, Osaka University Graduate School of Medicine, Osaka, Japan; b Cancer Control Center, Osaka International Cancer Institute, Osaka, Japan

**Keywords:** Tobacco, Heated tobacco product, Workplace smoking, Working hours

## Abstract

Although some private companies have implemented “time-based” workplace smoking bans covering all working hours including lunchtime, little is known about their impact on tobacco use. We examined associations between workplace smoking bans during working hours (including and excluding lunchtime) versus no ban and workers’ combustible cigarette and heated tobacco product (HTP) use.

We used data from the Japan “Society and New Tobacco” Internet Survey 2020 and in total 4,222 workers aged 20–74 were included in the analyses. Using inverse probability weighting, prevalence ratios (PRs) and 95% confidence interval (CI) for tobacco use were calculated according to “time-based” smoke-free policies: workplace smoking bans for working hours including lunchtime (lunchtime ban), workplace smoking ban for working hours excluding lunchtime (lunchtime allowed), and no ban for working hours (no ban).

The PRs of “lunchtime ban” for workers’ use of combustible cigarettes and HTPs were lower than “no ban”: 0.43 (CI: 0.29, 0.63) for combustible cigarette use and 0.61 (CI: 0.41, 0.90) for HTPs use. Meanwhile, the PRs for “lunchtime allowed” was not significant: 0.84 (CI: 0.70, 1.01) for combustible cigarettes use and 1.15 (CI: 0.94, 1.40) for HTPs use.

Workplace bans on smoking during working hours which include lunchtime may successfully reduce workers’ combustible cigarette and HTP use, but allowing their use during lunchtime may reduce the effectiveness of the ban. Not only conventional “place-based” smoke-free policies, but also “time-based” smoke-free policies are useful for tobacco control in the workplace.

## Highlights

• In some private companies, “time-based” workplace policies which ban smoking during working hours (including or excluding lunchtime) have been implemented.
• Little is known about the relationship between time-based smoke-free policies for working hours and combustible cigarette and HTP use. In this study, we focused on workplace bans on smoking during working hours including or excluding lunchtime.
• Bans on smoking during working hours which include lunchtime may be successful in reducing use of combustible cigarettes and HTPs, but allowing their use during lunchtime may reduce the effectiveness of the ban. Not only place-based, but also time-based smoke-free policies are important for tobacco control in the workplace.

## 1 Introduction

Tobacco use is a significant public health problem, with 7 million people worldwide dying annually from tobacco use and 1.2 million dying from passive smoking (Global Burden of Disease, 2019). To reduce smoking rates, the World Health Organization established the framework convention on tobacco control in 2005, suggesting various effective tobacco control measures such as tobacco taxation, smoke-free policies, and anti-tobacco media campaigns (Joossens and Raw, 2006). Smoke-free policies have been promoted to reduce smoking prevalence and prevent secondhand smoking. Previous studies have confirmed that passive smoking in the workplace was associated with many kinds of diseases such as cardiovascular disease (Bruckman and Bennett, 2011:7., Kent et al., 2012), respiratory disease (Dove et al., 2010), and low fertility rates (Kabir et al., 2009).

Regarding workplace smoke-free policies, previous studies have focused on place-based smoke-free policies such as a complete indoor smoking ban, partial indoor smoking ban or no ban. However, as the problem of staff leaving the workplace in order to smoke during working hours persists, time-based smoke-free policies such as a ban on smoking during hours worked were implemented by some companies in Japan (More and more companies are asking people to quit smoking while working at home, 2021). Furthermore, some of these companies have banned smoking during lunchtime in addition to working hours.

The impact of place-based smoke-free policies on smoking behavior has already been extensively examined (Working Procedures, 2009); for example, complete smoke-free policies in the workplace have been found to significantly reduce the prevalence of smoking compared to not having a smoking ban (Tabuchi et al., 2016). However, no study has investigated the impact of time-based smoke-free policies such as a ban on smoking during all working hours or working hours excluding lunchtime. A previous study in Australia reported that 40 % of smokers left their workplace to smoke at lunchtime while 13 % left at least once a day during working hours (Borland et al., 1997); therefore, a lunchtime smoking ban may be effective to reduce the amount smoked or to encourage smokers to quit.

The smoking rate, which does not distinguish between heated tobacco products (HTPs) and cigarettes, was reported as 17.8 % among Japanese adults (Ministry of Health, Labour and Welfare. National Health and Nutrition Survey, 2018.). Recently, in addition to combustible cigarettes, HTPs have become popular in Japan (Odani and Tabuchi, 2022). More than 10 % of Japanese adults aged 15–74 years used HTPs either “almost every day” or “sometimes” in 2020.

Our objective was to examine associations between time-based smoke-free policies in the workplace for working hours including or excluding lunchtime and tobacco product use (combustible cigarettes and HTPs). Because the impact of the smoke-free policies may differ between cigarette smoking and HTP use, we examined the prevalence of combustible cigarette use, HTP use, dual use, and any tobacco use in this study. Also, since there can be confounding between time-based smoke-free policies and place-based smoke-free policies, we performed stratified analyses according to place-based smoke-free policies.

## 2 Methods

### 2.1 Data

**Supplementary figure 1:**
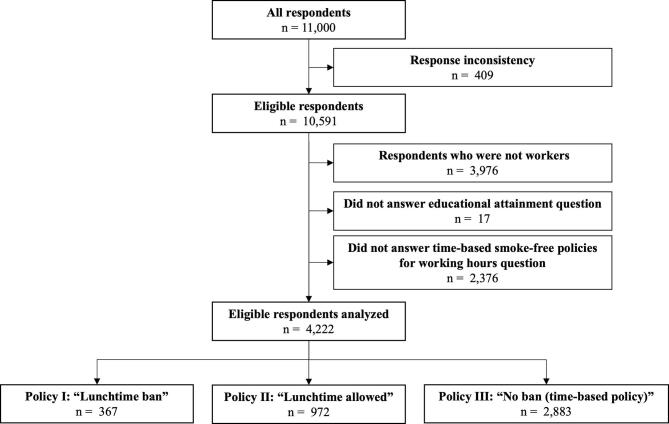
Study profile.

The Japan “Society and New Tobacco” Internet Survey (JASTIS) is a longitudinal internet-based cohort study designed to investigate the use of tobacco products: conventional cigarettes, HTPs and e-cigarettes. Details of the JASTIS have been described previously (Tabuchi et al., 2019). Participants in this survey are recruited from Rakuten Insight (Insight and Profiles, 2015), a nationwide internet survey agency with 2.3 million panelists covering various social categories, such as education, housing tenure and marital status, defined by the Japanese census. The JASTIS was launched in 2015 and an internet-based self-reported questionnaire survey is conducted once a year among panelists who are randomly selected and/or invited for follow-up. Each survey is closed when the target number of respondents who have answered the questionnaire is met. In this study, we used cross-sectional data from the survey conducted on 11,000 people in February 2020. Among the 11,000 respondents, 409 whose answers were inconsistent were excluded. A further 3,976 who were not workers were excluded, together with 17 who did not report their educational attainment, and 2,376 respondents who were working but did not answer the questions on time-based smoke-free policies for working hours (Supplementary Fig. 1).

### 2.2 Time-based smoke-free policies for working hours (policy I, II and III) (independent variables)

**Fig. 1:**
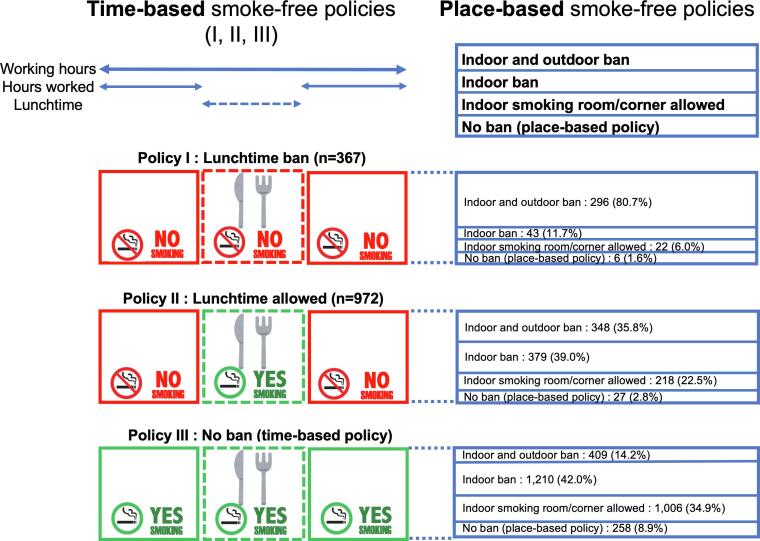
Time-based and place-based smoke-free policies.

The questionnaire offered three answer options to questions about workplace smoke-free policies for working hours. “Working hours” was defined as hours worked and lunchtime. Respondents who chose “in addition to hours worked, smoking is prohibited during lunchtime (no smoking during working hours)” were defined as “policy I: lunchtime ban”. Respondents who answered “workers must not smoke during hours worked but can smoke during lunchtime” were defined as “policy II: lunchtime allowed”. Respondents who answered “workers can smoke whenever they want during working hours” were defined as “policy III: no ban (time-based policy)” (Fig. 1).

### 2.3 Place-based smoke-free policies (confounding variables)

**Supplementary figure 2:**
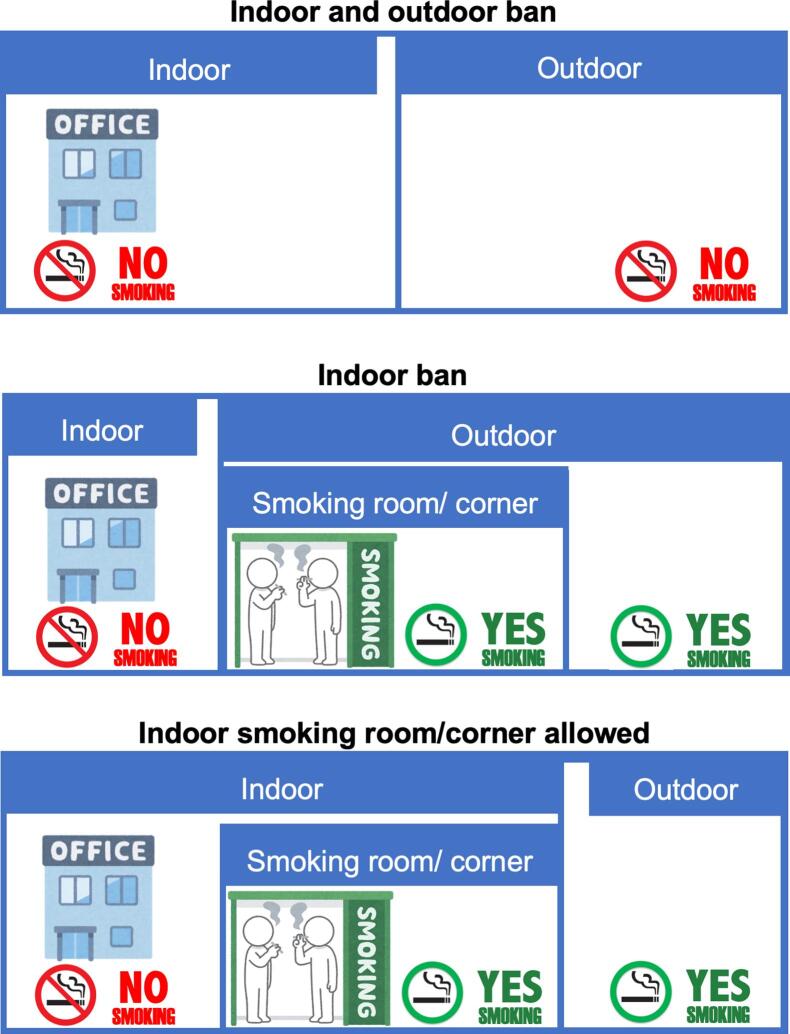
Description of place-based smoke-free policies on premises (indoor and outdoor).

Participants were asked about place-based smoke-free policies on their work premises. “Indoor and outdoor ban” was defined as no smoking in either indoor or outdoor locations in the workplace. “Indoor ban” was defined when indoor smoking was banned but a smoking room/corner was available outdoors in the workplace. “Indoor smoking room/corner allowed” was defined when indoor smoking was allowed in the smoking room/corner (Smoking rooms were closed off and smoking corners were open). “No ban (place-based policy)” was defined when smoking was not banned anywhere (Supplementary figure 2).

### 2.4 Definition of current tobacco product use (combustible cigarettes, HTPs and dual use) (dependent variables)

Participants were asked about their current use (regular use in the previous 30 days) of tobacco products. Respondents who answered “yes” to the question, “Have you used the following products in the previous 30 days?” (options: combustible cigarettes, IQOS, Ploom TECH, Ploom S, glo, glo sens, and PULZE) were defined as current users of the designated product (Giovenco et al., 2014, King et al., 2015). Current smokers of combustible cigarettes were defined as combustible cigarette current users. Current users of any of the following HTPs—IQOS, Ploom, glo or PULZE—were defined as current HTP users. Dual users were defined as current combustible cigarette users who had concurrently used any HTPs in the previous 30 days. Current users of either combustible cigarettes or HTPs were defined as any tobacco users.

### 2.5 Covariates (confounding variables)

Other variables used in this study were sex (man, woman), age group (20–29 years, 30–39, 40–49, 50–59, 60–74), employment status (company officer, regular employee, self-employed business owner, part-time contractor), industry (manufacturing, forestry, mining and construction, wholesale, retail trade, food and drink services, infrastructure, information and communications, finance, insurance, real estate, goods rental and leasing, medical, health care, welfare, education and public servant, other services). Covariates included educational attainment (high school or below, college, university, or graduate school), marital status (married, never married, divorced/widowed), housing (owns or does not own housing), and self-rated health (good or poor).

### 2.6 Statistical analysis

To observe the basic characteristics according to time-based smoke-free policies for working hours, differences in distribution of covariates, including socio-demographic factors, by time-based smoke-free policies were examined using the chi-squared test.

The prevalence ratios (PRs) and their 95 % confidence intervals (CIs) for current tobacco use were calculated by log-binomial regression models because most outcomes were not rare (>10 %) (Zhang and Yu, 1998, McNutt, 2003) (Supplementary table 1).

We used inverse probability of treatment weight (IPTW) to adjust for confounding variables. When assuming the effect of time-based smoke-free policies on dependent variables, we used the average treatment effect (ATE) (Dugoff et al., 2014). The reference category for time-based smoke-free policies was “no ban (time-based policy)” in table 2, and “lunchtime allowed” in supplementary table 2. To compare the effect of time-based smoke-free policies with place-based smoke-free policies, we used an independent log-binomial regression model for place-based smoke-free policies (supplementary table 3), because simultaneous input of both time-based and place-based policies might cause overadjustment. Stratified analyses were performed according to workplace place-based smoke-free policies to clarify the relationship between time-based smoke-free policies and place-based smoke-free policies (Table 3).

R (4.0.2) (R Core Team, 2020) was used for all analyses. The TWANG (1.6) (Ridgeway et al., 2020) package was used to calculate IPTW to compare three time-based smoke-free policies. To calculate weights for optimal balance, we used gradient boosted trees. The number of trees was set to 3,000, which was enough for convergence. Probability values for statistical tests were two tailed; p < 0.05 was considered statistically significant.

## 3 Results

A total of 4,222 workers were included in the analyses. The basic characteristics of study subjects are shown in Table 1. Regarding current tobacco use, 29.3 % used combustible cigarettes, 20.6 % used HTPs, 13.3 % used both cigarettes and HTPs (dual users), and 36.7 % used either cigarettes or HTPs (any tobacco use). Regarding time-based smoke-free policies for working hours, 8.7 % were “lunchtime ban”, 23.0 % were “lunchtime allowed”, and 68.3 % were “no ban (time-based policy)”. The prevalence of current HTPs use in “lunchtime ban”, “lunchtime allowed”, and “no ban (time-based policy)” were 11.7 %, 20.7 %, and 21.7 %, respectively. Regarding place-based smoke-free policies, 24.9 % were “indoor and outdoor ban”, 38.7 % were “indoor ban”, 29.5 % were “indoor smoking room/corner allowed”, and 6.9 % were “no ban (place-based policy)”. Distributions of sex, age, employment, industry, educational attainment, and place-based smoke-free policies differed significantly according to the time-based smoke-free policies (chi-square test).

**Table 1:** Basic characteristics according to time-based smoke-free policies for working hours.

		Time-based smoke-free policies for working hours			
Characteristics	Overall, N = 4,222^1^	Lunchtime ban, N = 367^1^	Lunchtime allowed, N = 972^1^	No ban (time-based policy), N = 2,883^1^	p-value^2^
**Sex**					**<0.001**
Man	2,934 (69.5 %)	219 (59.7 %)	613 (63.1 %)	2,102 (72.9 %)	
Woman	1,288 (30.5 %)	148 (40.3 %)	359 (36.9 %)	781 (27.1 %)	
**Age**					**<0.001**
20–29	729 (17.3 %)	74 (20.2 %)	194 (20.0 %)	461 (16.0 %)	
30–39	603 (14.3 %)	49 (13.4 %)	136 (14.0 %)	418 (14.5 %)	
40–49	1,077 (25.5 %)	80 (21.8 %)	222 (22.8 %)	775 (26.9 %)	
50–59	1,080 (25.6 %)	73 (19.9 %)	221 (22.7 %)	786 (27.3 %)	
60–74	733 (17.4 %)	91 (24.8 %)	199 (20.5 %)	443 (15.4 %)	
**Employment**					**<0.001**
Company officer	266 (6.3 %)	26 (7.1 %)	46 (4.7 %)	194 (6.7 %)	
Regular employee	2,580 (61.1 %)	184 (50.1 %)	519 (53.4 %)	1,877 (65.1 %)	
Self-employed business owner	411 (9.7 %)	61 (16.6 %)	80 (8.2 %)	270 (9.4 %)	
Part-time contractor	965 (22.9 %)	96 (26.2 %)	327 (33.6 %)	542 (18.8 %)	
**Industry**					**<0.001**
Manufacturing	800 (18.9 %)	44 (12.0 %)	234 (24.1 %)	522 (18.1 %)	
Forestry, mining and construction	300 (7.1 %)	15 (4.1 %)	39 (4.0 %)	246 (8.5 %)	
Wholesale, retail trade, food and drink services	558 (13.2 %)	39 (10.6 %)	149 (15.3 %)	370 (12.8 %)	
Infrastructure, information and communications	540 (12.8 %)	17 (4.6 %)	66 (6.8 %)	457 (15.9 %)	
Finance, insurance, real estate, goods rental and leasing	296 (7.0 %)	23 (6.3 %)	54 (5.6 %)	219 (7.6 %)	
Medical, health care, welfare, education and public servant	868 (20.6 %)	152 (41.4 %)	258 (26.5 %)	458 (15.9 %)	
Other services	860 (20.4 %)	77 (21.0 %)	172 (17.7 %)	611 (21.2 %)	
**Educational attainments**					**0.02**
High school or below	1,094 (25.9 %)	74 (20.2 %)	246 (25.3 %)	774 (26.8 %)	
College, university or graduate school	3,128 (74.1 %)	293 (79.8 %)	726 (74.7 %)	2,109 (73.2 %)	
**Marital status**					0.6
Married	2,386 (56.5 %)	218 (59.4 %)	533 (54.8 %)	1,635 (56.7 %)	
Never married	1,542 (36.5 %)	126 (34.3 %)	372 (38.3 %)	1,044 (36.2 %)	
Divorced or widowed	294 (7.0 %)	23 (6.3 %)	67 (6.9 %)	204 (7.1 %)	
**Housing**					0.3
Owns	1,433 (33.9 %)	120 (32.7 %)	311 (32.0 %)	1,002 (34.8 %)	
Does not own	2,789 (66.1 %)	247 (67.3 %)	661 (68.0 %)	1,881 (65.2 %)	
**Self-rated health**					0.7
Good	3,795 (89.9 %)	328 (89.4 %)	868 (89.3 %)	2,599 (90.1 %)	
Poor	427 (10.1 %)	39 (10.6 %)	104 (10.7 %)	284 (9.9 %)	
**Place-based smoke-free policies**					**<0.001**
Indoor and outdoor ban	1,053 (24.9 %)	296 (80.7 %)	348 (35.8 %)	409 (14.2 %)	
Indoor ban	1,632 (38.7 %)	43 (11.7 %)	379 (39.0 %)	1,210 (42.0 %)	
Indoor smoking room/corner allowed	1,246 (29.5 %)	22 (6.0 %)	218 (22.4 %)	1,006 (34.9 %)	
No ban (place-based policy)	291 (6.9 %)	6 (1.6 %)	27 (2.8 %)	258 (8.9 %)	
**Combustible cigarette use**					**<0.001**
Not current user	2,983 (70.7 %)	319 (86.9 %)	724 (74.5 %)	1,940 (67.3 %)	
Current user	1,239 (29.3 %)	48 (13.1 %)	248 (25.5 %)	943 (32.7 %)	
**HTP use**					**<0.001**
Not current user	3,352 (79.4 %)	324 (88.3 %)	771 (79.3 %)	2,257 (78.3 %)	
Current user	870 (20.6 %)	43 (11.7 %)	201 (20.7 %)	626 (21.7 %)	
**Dual use**					**<0.001**
Not current user	3,662 (86.7 %)	342 (93.2 %)	844 (86.8 %)	2,476 (85.9 %)	
Current user	560 (13.3 %)	25 (6.8 %)	128 (13.2 %)	407 (14.1 %)	
**Any tobacco use**					**<0.001**
Not current user	2,673 (63.3 %)	301 (82.0 %)	651 (67.0 %)	1,721 (59.7 %)	
Current user	1,549 (36.7 %)	66 (18.0 %)	321 (33.0 %)	1,162 (40.3 %)	

^1^n (%)

^2^Pearson's Chi-squared test

HTPs = Heated tobacco products

Table 2 shows PRs (95 % CI) by the log-binomial regression models for each current tobacco use according to time-based smoke-free policies with IPTW adjustments for potential covariates. Regarding combustible cigarette use, PRs (95 % CI) for “lunchtime ban” and “lunchtime allowed” were 0.4 (0.29, 0.63) and 0.84 (0.70, 1.01), respectively. For HTP use, PRs for “lunchtime ban” and “lunchtime allowed” were 0.61 (0.41, 0.90) and 1.15 (0.94, 1.40), respectively. For dual use, PRs for “lunchtime ban” and “lunchtime allowed” were 0.57 (0.35, 0.94) and 1.16 (0.92, 1.47), respectively. For any tobacco use, PRs for “lunchtime ban” and “lunchtime allowed” were 0.44 (0.31, 0.62) and 0.88 (0.74, 1.04), respectively.

**Table 2:** Prevalence ratios (95% CI) for combustible cigarette and HTP use according to time-based smoke-free policies by log-binomial regression model with IPTW adjustments for potential covariates.

	Overall	Combustible cigarette use	HTP use	Dual use	Any tobacco use	Maximum standardized difference*	
Characteristics	N (%)	PR^1^ (95 % CI)	PR^1^ (95 % CI)	PR^1^ (95 % CI)	PR^1^ (95 % CI)	Before	After
**Time-based smoke-free policies**							
Lunchtime ban	367 (9 %)	**0.43 (0.29, 0.63)**	**0.61 (0.41, 0.90)**	**0.57 (0.35, 0.94)**	**0.44 (0.31, 0.62)**		
Lunchtime allowed	972 (23 %)	0.84 (0.70, 1.01)	1.15 (0.94, 1.40)	1.16 (0.92, 1.47)	0.88 (0.74, 1.04)		
No ban (time-based policy)	2883 (68 %)	1.00 (reference)	1.00 (reference)	1.00 (reference)	1.00 (reference)		
**Sex**							
Man	2934 (69 %)	1.00 (reference)	1.00 (reference)	1.00 (reference)	1.00 (reference)	0.234	0.027
Woman	1288 (31 %)	**0.47 (0.35, 0.64)**	**0.57 (0.42, 0.78)**	**0.64 (0.43, 0.95)**	**0.45 (0.35, 0.59)**	0.234	0.027
**Age**							
20–29	729 (17 %)	1.00 (reference)	1.00 (reference)	1.00 (reference)	1.00 (reference)	0.106	0.017
30–39	603 (14 %)	**1.99 (1.13, 3.50)**	0.97 (0.60, 1.58)	1.24 (0.62, 2.46)	1.49 (0.95, 2.36)	0.029	0.02
40–49	1077 (26 %)	**2.74 (1.62, 4.63)**	0.75 (0.48, 1.15)	1.26 (0.70, 2.28)	**1.60 (1.05, 2.43)**	0.099	0.006
50–59	1080 (26 %)	**3.31 (1.86, 5.91)**	0.78 (0.47, 1.29)	1.27 (0.64, 2.53)	**1.95 (1.22, 3.12)**	0.143	0.027
60–74	733 (17 %)	**2.79 (1.55, 5.01)**	**0.56 (0.33, 0.97)**	1.25 (0.61, 2.56)	1.35 (0.83, 2.18)	0.215	0.024
**Employment**							
Company officer	266 (6 %)	1.00 (reference)	1.00 (reference)	1.00 (reference)	1.00 (reference)	0.084	0.042
Regular employee	2580 (61 %)	1.39 (0.95, 2.03)	1.00 (0.67, 1.49)	1.45 (0.90, 2.33)	1.09 (0.75, 1.58)	0.259	0.015
Self-employed business owner	411 (10 %)	**1.61 (1.06, 2.46)**	0.79 (0.50, 1.24)	1.11 (0.64, 1.93)	1.21 (0.80, 1.83)	0.254	0.009
Part-time contractor	965 (23 %)	1.09 (0.72, 1.65)	**0.56 (0.36, 0.87)**	0.65 (0.38, 1.13)	0.86 (0.58, 1.27)	0.334	0.028
**Industry**							
Manufacturing	800 (19 %)	1.00 (reference)	1.00 (reference)	1.00 (reference)	1.00 (reference)	0.194	0.035
Forestry, mining and construction	300 (7 %)	0.97 (0.57, 1.63)	1.20 (0.68, 2.12)	1.04 (0.53, 2.02)	1.09 (0.65, 1.82)	0.175	0.035
Wholesale, retail trade, food and drink services	558 (13 %)	0.78 (0.49, 1.23)	0.90 (0.58, 1.39)	0.74 (0.42, 1.30)	0.86 (0.57, 1.28)	0.084	0.013
Infrastructure, information and communications	540 (13 %)	0.92 (0.56, 1.50)	1.21 (0.76, 1.95)	1.11 (0.62, 2.00)	1.02 (0.65, 1.60)	0.289	0.097
Finance, insurance, real estate, goods rental and leasing	296 (7 %)	0.68 (0.42, 1.09)	0.89 (0.54, 1.46)	0.65 (0.37, 1.12)	0.81 (0.52, 1.27)	0.074	0.006
Medical, health care, welfare, education and public servant	868 (21 %)	**0.65 (0.47, 0.90)**	0.82 (0.57, 1.18)	0.73 (0.47, 1.11)	**0.71 (0.52, 0.96)**	0.565	0.054
Other services	860 (20 %)	0.79 (0.57, 1.10)	0.93 (0.62, 1.37)	0.90 (0.59, 1.39)	0.81 (0.58, 1.14)	0.086	0.007
**Educational attainments**							
High school or below	1094 (26 %)	1.00 (reference)	1.00 (reference)	1.00 (reference)	1.00 (reference)	0.144	0.03
College, university or graduate school	3128 (74 %)	0.90 (0.71, 1.15)	0.84 (0.63, 1.11)	0.91 (0.67, 1.24)	0.84 (0.66, 1.07)	0.144	0.03
**Marital status**							
Married	2386 (57 %)	1.00 (reference)	1.00 (reference)	1.00 (reference)	1.00 (reference)	0.064	0.067
Never married	1542 (37 %)	1.31 (0.93, 1.83)	**0.70 (0.51, 0.98)**	0.89 (0.58, 1.35)	1.03 (0.77, 1.40)	0.05	0.017
Divorced or widowed	294 (7 %)	**1.60 (1.10, 2.32)**	1.35 (0.91, 2.01)	1.28 (0.80, 2.05)	**1.65 (1.16, 2.35)**	0.03	0.098
**Housing**							
Owns	1433 (34 %)	1.00 (reference)	1.00 (reference)	1.00 (reference)	1.00 (reference)	0.054	0.039
Does not own	2789 (66 %)	1.13 (0.88, 1.45)	1.18 (0.89, 1.56)	1.37 (0.97, 1.93)	1.06 (0.84, 1.34)	0.054	0.039
**Self-rated health**							
Good	3795 (90 %)	1.00 (reference)	1.00 (reference)	1.00 (reference)	1.00 (reference)	0.027	0.035
Poor	427 (10 %)	0.97 (0.70, 1.35)	1.03 (0.74, 1.43)	0.87 (0.60, 1.26)	1.06 (0.78, 1.45)	0.027	0.035

^1^PR = Prevalence Ratio, CI = Confidence Interval^.^

*Maximum standardized pairwise difference, before and after inverse probability of treatment weighting.

**Table 3:** Stratified analyses among place-based smoke-free policies for combustible cigarette and HTP use according to time-based smoke-free policies by inverse probability of treatment weight.

Smoke-free policies		N = 3,931^†^	Combustible cigarette use	HTP use	Dual use	Any tobacco use
**Place-based smoke-free policies**	**Time-based smoke-free policies**	N (%)	PR^1^ (95 % CI)	PR^1^ (95 % CI)	PR^1^ (95 % CI)	PR^1^ (95 % CI)
Indoor and outdoor ban	Lunchtime ban	296 (28 %)	**0.55 (0.35, 0.88)**	**0.52 (0.30, 0.90)**	0.63 (0.33, 1.19)	**0.49 (0.31, 0.75)**
	Lunchtime allowed	348 (33 %)	0.82 (0.56, 1.21)	1.13 (0.74, 1.72)	1.15 (0.69, 1.90)	0.86 (0.60, 1.22)
	No ban (time-based policy)	409 (39 %)	1.00 (reference)	1.00 (reference)	1.00 (reference)	1.00 (reference)
Indoor ban	Lunchtime ban	43 (3 %)	**0.28 (0.10, 0.81)**	**2.15 (1.003, 4.59)**	0.77 (0.25, 2.42)	0.81 (0.39, 1.71)
	Lunchtime allowed	379 (23 %)	1.04 (0.76, 1.42)	**1.45 (1.01, 2.09)**	1.25 (0.82, 1.88)	1.22 (0.90, 1.66)
	No ban (time-based policy)	1210 (74 %)	1.00 (reference)	1.00 (reference)	1.00 (reference)	1.00 (reference)
Indoor smoking room/corner allowed	Lunchtime ban	22 (2 %)	0.79 (0.31, 2.04)	1.83 (0.75, 4.46)	1.31 (0.43, 3.94)	1.16 (0.46, 2.90)
	Lunchtime allowed	218 (17 %)	0.99 (0.66, 1.49)	1.20 (0.78, 1.86)	1.41 (0.87, 2.29)	0.93 (0.63, 1.38)
	No ban (time-based policy)	1006 (81 %)	1.00 (reference)	1.00 (reference)	1.00 (reference)	1.00 (reference)

^†^ Total N: Among 4,222 respondents, 291 who answered “no ban (place-based policy)” for place-based smoke-free policies were excluded.

^1^PR = Prevalence Ratio, CI = Confidence Interval

Among covariates, in the “sex” category, women had significantly lower PRs for all four tobacco use categories. In the “age” category, for combustible cigarette use, the PRs for the 30–74 years group were higher than the 20–29 years group. For HTP use, there was no statistically significant difference between the 30–59 years group and the 20–29 years group, but the 60–74 years group was significantly lower than the 20–29 years group.

Even when the reference category for time-based smoke-free policies was changed to “lunchtime allowed”, PRs for all four tobacco use outcomes were significantly lower than one (supplementary table 2).

Results stratified by place-based smoke-free policies at workplace are shown in Table 3. For combustible cigarettes, PRs of “lunchtime ban” and “lunchtime allowed” within “indoor and outdoor ban” were 0.55 (0.35, 0.88) and 0.82 (0.56, 1.21), respectively. PRs of “lunchtime ban” and “lunchtime allowed” within “indoor ban” were 0.28 (0.10, 0.81) and 1.04 (0.76, 1.42), respectively.

For HTP use, PRs of “lunchtime ban” and “lunchtime allowed” within “indoor and outdoor ban” were 0.52 (0.30, 0.90) and 1.13 (0.74, 1.72), respectively. The PRs of “lunchtime ban” and “lunchtime allowed” within “indoor ban” were 2.15 (1.003, 4.59) and 1.45 (1.01, 2.09), respectively.

## 4 Discussion

To our best knowledge, this is the first study to examine the effects of workplace smoking bans during working hours on smoking prevalence. We compared three kinds of time-based smoke-free policies: “working hours including lunchtime ban (lunchtime ban)”, “ban during working hours, but lunchtime allowed”, and “no ban (time-based policy)”. Workers were 57 % less likely to use combustible cigarettes when workplace smoking was banned during working hours including lunchtime (lunchtime ban) than “no ban (time-based policy)”. HTP use was 39 % lower, and dual use was 43 % lower in “lunchtime ban” compared with “no ban (time-based policy)”. Furthermore, when workers were not allowed to smoke during working hours but could smoke at lunchtime (“lunchtime allowed”), workers were less likely to use combustible cigarettes (point estimate of PR = 0.84), but rather more likely to use HTPs (point estimate of PR = 1.15) compared with “no ban (time-based policy)”, although these PRs were not statistically significant. Workplace smoking bans for all working hours including lunchtime may be a useful additional key tobacco control measure in future.

The impact of the workplace smoking ban for working hours may be different in combination with place-based smoke-free policies (“only indoor ban” or “indoor and outdoor ban”): for example, compared with “no ban (time-based policy)”, combustible cigarette use was significantly lower in “lunchtime ban” within both “indoor and outdoor ban” and “indoor ban”, but HTP use was 48 % lower within “indoor and outdoor ban” while inversely it was 115 % higher within “indoor ban”. Furthermore, compared with “no ban”, HTP use was 45 % higher in “lunchtime allowed” within “indoor ban”. When smokers can smoke outdoors, they may be more likely to use HTPs than cigarettes. For “lunchtime ban” with “indoor ban” and “indoor smoking room/corner allowed”, these percentages were small; only 3 % and 2 % of each place-based smoke-free policy respectively. It has previously been suggested that the advent of HTP use may weaken the impact of smoke-free policies (Tabuchi, 2021), as we have observed in the current study.

Although some private companies in Japan have begun to establish time-based smoke-free policies during working hours, there is, as yet, no scientific evidence to enable us to evaluate the interactions between time-based and place-based policies.

For future research, this analysis of time-based smoke-free policies could be extended to include similar efforts in schools as well as workplaces. For example, smoke-free policies where attention may shift from place to time e.g. from “smoke-free school premises” to “smoke-free school hours” (Jakobsen et al., 2021). Consideration of “time-based” smoke-free policies would add a new level to traditional smoke-free policies.

### 4.1 Limitations

There were several limitations in this study. First, because the results were obtained from an internet survey, sample distribution may differ from population-based studies in categories such as higher educational attainments. In addition, it was a self-report survey and did not measure objective indicators such as blood cotinine for smoking status. Further, responses may have been based on the employee's understanding of the workplace policy rather than the actual policy. However, previous studies have shown that the reliability of self-reporting smoking behavior is generally high (Caraballo et al., 2001).

Second, because of the cross-sectional design of the study, estimation of causal inference was difficult. We observed relationships between time-based smoke-free policies at workplaces and combustible cigarettes and HTPs use. We cannot surmise whether smoking bans affect smoking habits, smoking habits in each group affect smoking bans, or both. To overcome this, a longitudinal study of the interrelationship between time-based and place-based policies is needed in the future.

Finally, the sample size of “lunchtime ban” was small. The sample size of stratified analyses may not be great enough for statistical significance. This is probably because the time-based smoke-free policies for working hours have not received as much attention as the place-based smoke-free policies. This low prevalence of “lunchtime ban” represents a continuing issue for future tobacco control efforts.

## 5 Conclusions

To date, little is known about smoking policies for working hours and their effect on tobacco use. Not only place-based, but also time-based smoke-free policies are important for tobacco control in the workplace. Smoking bans for working hours including lunchtime may successfully reduce the use of combustible cigarettes and HTPs, but allowing their use during lunchtime may reduce the effectiveness of the ban.

## 6 Ethics approval

The study was reviewed and approved by the Research Ethics Committee of the Osaka International Cancer Institute (no. 1412175183).

## 7 Data availability statement

Data are available on reasonable request. The JASTIS data can be accessed via the corresponding author, TT, on reasonable request.

## Funding

This work was supported by Japan Society for the Promotion of Science (JSPS) KAKENHI Grants (JP21H04856).

## CRediT authorship contribution statement

**Yuki Miyazaki:** Conceptualization, Formal analysis, Investigation, Methodology, Software, Validation, Visualization, Writing – original draft. **Takahiro Tabuchi:** Data curation, Funding acquisition, Project administration, Resources, Supervision, Writing – review & editing.

## Declaration of Competing Interest

The authors declare that they have no known competing financial interests or personal relationships that could have appeared to influence the work reported in this paper.

## Data Availability

Data will be made available on request.

## References

[b0005] Borland R., Cappiello M., Owen N. (1997). Leaving work to smoke. Addiction.

[b0010] Bruckman D., Bennett B. (2011). Significant change in statewide rates of hospital discharge data for myocardial infarction due to the enactment of Ohio's Smoke Free Workplace Law. Analyses of the Impact of the Ohio Smoke Free Workplace Act. Ohio Department of. Health.

[b0015] Caraballo R.S., Giovino G.A., Pechacek T.F., Mowery P.D. (2001). Factors associated with discrepancies between self-reports on cigarette smoking and measured serum cotinine levels among persons aged 17 years or older: Third National Health and Nutrition Examination Survey, 1988–1994. Am. J. Epidemiol..

[b0020] Dove M.S., Dockery D.W., Mittleman M.A., Schwartz J., Sullivan E.M., Keithly L., Land T. (2010). The impact of Massachusetts' smoke-free workplace laws on acute myocardial infarction deaths. Am. J. Public Health.

[b0025] Dugoff E.H., Schuler M., Stuart E.A. (2014). Generalizing observational study results: applying propensity score methods to complex surveys. Health Serv. Res..

[b0030] Giovenco D.P., Lewis M.J., Delnevo C.D. (2014). Factors associated with e-cigarette use: a national population survey of current and former smokers. Am. J. Prev. Med..

[b0035] Global Burden of Disease [database]. Washington, DC: Institute of Health Metrics; 2019. IHME, accessed 17 July 2021.

[b0040] Insight R, Profiles M, 2015. Available: https://insight.rakuten.co.jp/en/ [Accessed 1 September 2021].

[b0045] Jakobsen G., Danielsen D., Jensen M., Vinther J., Pisinger C., Holmberg T., Krølner R., Andersen S. (2021). “Reducing smoking in youth by a smoke-free school environment: A stratified cluster randomized controlled trial of Focus, a multicomponent program for alternative high schools. Tobacco Prevent. Cessation.

[b0050] Joossens L., Raw M. (2006). The Tobacco Control Scale: a new scale to measure country activity. Tob. Control.

[b0055] Kabir Z., Clarke V., Conroy R., McNamee E., Daly S., Clancy L. (2009). Low birthweight and preterm birth rates 1 year before and after the Irish workplace smoking ban. Br. J. Obstet. Gynaecol..

[b0060] Kent B.D., Sulaiman I., Nicholson T.T., Lane S.J., Moloney E.D. (2012). Acute pulmonary admissions following implementation of a national workplace smoking ban. Chest.

[b0065] King B.A., Patel R., Nguyen K.H., Dube S.R. (2015). Trends in awareness and use of electronic cigarettes among US adults, 2010–2013. Nicotine Tob. Res..

[b0070] McNutt L.-A., Chuntao Wu, Xue Xiaonan, Hafner Jean Paul (2003). Estimating the Relative Risk in Cohort Studies and Clinical Trials of Common Outcomes. Am. J. Epidemiol..

[15] Ministry of Health, Labour and Welfare. National Health and Nutrition Survey, 2018. Available at : https://www.nibiohn.go.jp/eiken/kenkounippon21/download_files/eiyouchousa/2018.pdf (accessed 6 June 2022).

[b0080] “More and more companies are asking people to quit smoking while working at home,” Japan Broadcasting Corporation, accessed 1 September 2021, from https://www3.nhk.or.jp/news/html/20210901/k10013235821000.html.

[b0085] Odani S., Tabuchi T. (2022). Prevalence of heated tobacco product use in Japan: the 2020 JASTIS study. Tob. Control.

[b0090] R Core Team, 2020. R: A language and environment for statistical computing. R Foundation for Statistical Computing, Vienna, Austria.

[b0095] Greg Ridgeway, Dan McCaffrey, Andrew Morral, Beth Ann Griffin, Lane Burgette and Matthew Cefalu, 2020. twang: Toolkit for Weighting and Analysis of Nonequivalent Groups. R package version 1.6. https://cran.r-project.org/web/packages/twang/vignettes/twang.pdf.

[b0100] Tabuchi T. (2021).

[b0105] Tabuchi T., Hoshino T., Nakayama T. (2016). Are Partial Workplace Smoking Bans as Effective as Complete Smoking Bans? A National Population-Based Study of Smoke-Free Policy Among Japanese Employees. Nicotine Tobacco Res..

[b0110] Tabuchi T., Shinozaki T., Kunugita N., Nakamura M., Tsuji I. (2019). Study Profile: The Japan ‘Society and New Tobacco’ Internet Survey (JASTIS): A Longitudinal Internet Cohort Study of Heat-Not-Burn Tobacco Products, Electronic Cigarettes, and Conventional Tobacco Products in Japan. J. Epidemiol..

[b0115] Working Procedures in: IARC Handbooks of Cancer Prevention: Tobacco Control. Volume 13. Evaluating the Effectiveness of Smoke-free Policies. Lyon, France: International Agency for Research on Cancer, 2009:327-334.

[b0120] Zhang J., Yu K.F. (1998). “What’s the Relative Risk? A Method of Correcting the Odds Ratio in Cohort Studies of Common Outcomes. JAMA: J. Am. Medical Assoc..

