# Supplementary material for: “Time-based” workplace smoking bans during working hours (including and excluding lunchtime) and combustible cigarette and heated tobacco product use: a cross-sectional analysis of the 2020 JASTIS study
Source: Prev Med Rep. 2022 Jul 29;29:101938. doi: 10.1016/j.pmedr.2022.101938 (PMC9356268; doi:10.1016/j.pmedr.2022.101938)
Supplement: Supplementary data 1 [file mmc1.docx]

Supplementary table 1. Numbers and percentages for combustible cigarette and HTP use according to characteristics.

|  | Overall | Combustible cigarette use | HTP use | Dual use | Any tobacco use |
| --- | --- | --- | --- | --- | --- |
| Characteristics | N (%) | N (%) | N (%) | N (%) | N (%) |
| Total | 4222 (100.0%) | 1239 (29.3%) | 870 (20.6%) | 560 (13.3%) | 1549 (36.7%) |
| Time-based smoke-free policies |  | * | * | * | * |
| No ban (time-based policy) | 2883 (68.3%) | 943 (32.7%) | 626 (21.7%) | 407 (14.1%) | 1162 (40.3%) |
| Lunchtime allowed | 972 (23.0%) | 248 (25.5%) | 201 (20.7%) | 128 (13.2%) | 321 (33.0%) |
| Lunchtime ban | 367 (8.7%) | 48 (13.1%) | 43 (11.7%) | 25 (6.8%) | 66 (18.0%) |
| Sex |  | * | * | * | * |
| Man | 2934 (69.5%) | 1035 (35.3%) | 723 (24.6%) | 469 (16.0%) | 1289 (43.9%) |
| Woman | 1288 (30.5%) | 204 (15.8%) | 147 (11.4%) | 91 (7.1%) | 260 (20.2%) |
| Age |  | * | * | * | * |
| 20-29 | 729 (17.3%) | 93 (12.8%) | 122 (16.7%) | 63 (11.3%) | 152 (20.9%) |
| 30-39 | 603 (14.3%) | 148 (24.5%) | 121 (20.1%) | 75 (12.4%) | 194 (32.2%) |
| 40-49 | 1077 (25.5%) | 342 (31.8%) | 242 (22.5%) | 156 (14.5%) | 428 (39.7%) |
| 50-59 | 1080 (25.6%) | 410 (38.0%) | 253 (23.4%) | 167 (15.5%) | 496 (45.9%) |
| 60-74 | 733 (17.4%) | 246 (33.6%) | 132 (18.0%) | 99 (13.5%) | 279 (38.1%) |
| Employment |  | * | * | * | * |
| Company officer | 266 (6.3%) | 91 (34.2%) | 81 (30.5%) | 40 (15.0%) | 132 (49.6%) |
| Regular employees | 2580 (61.1%) | 748 (29.0%) | 564 (21.9%) | 375 (14.5%) | 937 (36.3%) |
| Self-employed business owner | 411 (9.7%) | 189 (46.0%) | 95 (23.1%) | 68 (16.5%) | 216 (52.6%) |
| Part-time contractor | 965 (22.9%) | 211 (21.9%) | 130 (13.5%) | 77 (8.0%) | 264 (27.4%) |
| Industry |  | * | * | * | * |
| Manufacturing | 800 (18.9%) | 256 (32.0%) | 173 (21.6%) | 120 (15.0%) | 309 (38.6%) |
| Forestry, mining and construction | 300 (7.1%) | 104 (34.7%) | 79 (26.3%) | 50 (16.7%) | 133 (44.3%) |
| Wholesale, retail trade, eating and drinking services | 558 (13.2%) | 150 (26.9%) | 109 (19.5%) | 62 (11.1%) | 197 (35.3%) |
| Infrastructure, information and communications | 540 (12.8%) | 165 (30.6%) | 124 (23.0%) | 73 (13.5%) | 216 (40.0%) |
| Finance, insurance, real estate, goods rental and leasing | 296 (7.0%) | 83 (28.0%) | 64 (21.6%) | 38 (12.8%) | 109 (36.8%) |
| Medical, health care, welfare, education and public servant | 868 (20.6%) | 190 (21.9%) | 149 (17.2%) | 93 (10.7%) | 246 (28.3%) |
| Other services | 860 (20.4%) | 291 (33.8%) | 172 (19.8%) | 124 (14.4%) | 339 (39.4%) |
| Educational attainment |  | * |  |  | * |
| High school or below | 1094 (25.9%) | 361 (33.0%) | 235 (21.5%) | 150 (13.7%) | 446 (40.8%) |
| College, university or graduate school | 3128 (74.1%) | 878 (28.1%) | 635 (20.3%) | 410 (13.1%) | 1103 (35.3%) |
| Marital status |  | * | * | * | * |
| Married | 2386 (56.5%) | 736 (30.8%) | 533 (22.3%) | 340 (14.2%) | 929 (38.9%) |
| Never Married | 1542 (36.5%) | 395 (25.6%) | 264 (17.1%) | 177 (11.5%) | 482 (31.3%) |
| Divorced or widowed | 294 (7.0%) | 108 (36.7%) | 73 (24.8%) | 43 (14.6%) | 138 (46.9%) |
| Housing |  | * |  | * |  |
| Owns | 1433 (33.9%) | 379 (26.4%) | 278 (19.4%) | 153 (10.7%) | 504 (35.2%) |
| Dose not own | 2789 (66.1%) | 860 (30.8%) | 592 (21.2%) | 407 (14.6%) | 1045 (37.5%) |
| Self-rated health |  |  |  |  |  |
| Good | 3795 (89.9%) | 1110 (29.2%) | 766 (20.2%) | 500 (13.2%) | 1376 (36.3%) |
| Poor | 427 (10.1%) | 129 (30.2%) | 104 (24.4%) | 60 (14.1%) | 173 (40.5%) |
| HTP: heated tobacco product, Dual use: both combustible cigarettes and HTPs.  *statistically significant p<0.05 by chi-square test |  |  |  |  |  |

Supplementary table 2. Reference: “lunchtime allowed”.

|  | Overall | Combustible cigarette use | HTP use | Dual use | Any tobacco use |
| --- | --- | --- | --- | --- | --- |
| Characteristics | N (%) | PR^1^ (95% CI) | PR^1^ (95% CI) | PR^1^ (95% CI) | PR^1^ (95% CI) |
| **Time-based smoke-free policies** |  |  |  |  |  |
| Lunchtime ban | 367 (9%) | **0.41 (0.29, 0.58)** | **0.50 (0.35, 0.72)** | **0.48 (0.30, 0.74)** | **0.41 (0.30, 0.56)** |
| Lunchtime allowed | 972 (23%) | 1.00 (reference) | 1.00 (reference) | 1.00 (reference) | 1.00 (reference) |
| No ban (time-based policy) | 2883 (68%) | **1.23 (1.03, 1.46)** | 0.89 (0.74, 1.07) | 0.92 (0.74, 1.16) | 1.15 (0.97, 1.35) |
| Adjusted for sex, age, employment, industry, education, marital status, housing, and self-rated health. | | | | | |
| ^1^PR = Prevalence Ratio, CI = Confidence Interval | | | | | |

Supplementary table 3. Prevalence ratios (95% CI) for combustible cigarette and HTP use according to place-based smoke-free policies by log-binomial regression model.

|  | Overall | Combustible cigarette use | HTP use | Dual use | Any tobacco use | Maximum standardized difference* | |
| --- | --- | --- | --- | --- | --- | --- | --- |
| Characteristics | N (%) | PR^1^ (95% CI) | PR^1^ (95% CI) | PR^1^ (95% CI) | PR^1^ (95% CI) | Before | After |
| **Place-based** |  |  |  |  |  |  |  |
| **smoke-free policies** |  |  |  |  |  |  |  |
| Indoor and outdoor ban | 1053 (25%) | 1.00 (reference) | 1.00 (reference) | 1.00 (reference) | 1.00 (reference) |  |  |
| Indoor ban | 1632 (39%) | **1.85 (1.47, 2.32)** | **1.58 (1.22, 2.03)** | **1.38 (1.02, 1.85)** | **2.03 (1.64, 2.52)** |  |  |
| Indoor smoking room/corner allowed | 1246 (30%) | **2.22 (1.73, 2.85)** | **2.17 (1.64, 2.86)** | **1.80 (1.31, 2.49)** | **2.65 (2.09, 3.36)** |  |  |
| No ban (place-based policy) | 291 (7%) | 1.08 (0.72, 1.63) | 1.21 (0.76, 1.94) | 0.63 (0.34, 1.17) | **1.48 (1.01, 2.17)** |  |  |
| **Sex** |  |  |  |  |  |  |  |
| Man | 2934 (69%) | 1.00 (reference) | 1.00 (reference) | 1.00 (reference) | 1.00 (reference) | 0.197 | 0.048 |
| Woman | 1288 (31%) | **0.41 (0.33, 0.53)** | **0.42 (0.32, 0.56)** | **0.45 (0.33, 0.62)** | **0.37 (0.29, 0.47)** | 0.197 | 0.048 |
| **Age** |  |  |  |  |  |  |  |
| 20-29 | 729 (17%) | 1.00 (reference) | 1.00 (reference) | 1.00 (reference) | 1.00 (reference) | 0.071 | 0.096 |
| 30-39 | 603 (14%) | **2.46 (1.65, 3.66)** | 1.26 (0.82, 1.96) | 1.41 (0.86, 2.31) | **2.06 (1.41, 3.01)** | 0.143 | 0.025 |
| 40-49 | 1077 (26%) | **2.68 (1.87, 3.86)** | 0.80 (0.56, 1.15) | 1.28 (0.82, 2.02) | **1.64 (1.18, 2.27)** | 0.083 | 0.065 |
| 50-59 | 1080 (26%) | **3.68 (2.53, 5.35)** | 0.98 (0.66, 1.46) | 1.59 (0.98, 2.59) | **2.28 (1.62, 3.20)** | 0.106 | 0.006 |
| 60-74 | 733 (17%) | **2.98 (1.96, 4.54)** | **0.52 (0.35, 0.79)** | 1.15 (0.70, 1.89) | 1.42 (0.97, 2.08) | 0.074 | 0.149 |
| **Employment** |  |  |  |  |  |  |  |
| Company officer | 266 (6%) | 1.00 (reference) | 1.00 (reference) | 1.00 (reference) | 1.00 (reference) | 0.096 | 0.011 |
| Regular employee | 2580 (61%) | 0.84 (0.56, 1.25) | **0.63 (0.42, 0.94)** | 1.13 (0.70, 1.84) | **0.55 (0.37, 0.81)** | 0.498 | 0.036 |
| Self-employed business owner | 411 (10%) | **1.68 (1.07, 2.63)** | 0.71 (0.45, 1.13) | 1.23 (0.69, 2.19) | 1.12 (0.73, 1.73) | 1.104 | 0.078 |
| Part-time contractor | 965 (23%) | **0.59 (0.37, 0.95)** | **0.44 (0.28, 0.70)** | **0.60 (0.34, 1.06)** | **0.42 (0.27, 0.65)** | 0.145 | 0.011 |
| **Industry** |  |  |  |  |  |  |  |
| Manufacturing | 800 (19%) | 1.00 (reference) | 1.00 (reference) | 1.00 (reference) | 1.00 (reference) | 0.35 | 0.071 |
| Forestry, mining and construction | 300 (7%) | 1.25 (0.83, 1.88) | 1.30 (0.84, 2.02) | 1.22 (0.76, 1.93) | 1.35 (0.89, 2.04) | 0.191 | 0.039 |
| Wholesale, retail trade, food and drink services | 558 (13%) | 1.05 (0.75, 1.46) | 1.10 (0.75, 1.61) | 0.99 (0.64, 1.52) | 1.13 (0.81, 1.57) | 0.311 | 0.044 |
| Infrastructure, information and communications | 540 (13%) | 0.91 (0.63, 1.32) | 1.20 (0.78, 1.84) | 0.82 (0.54, 1.25) | 1.18 (0.82, 1.71) | 0.261 | 0.012 |
| Finance, insurance, real estate, goods rental and leasing | 296 (7%) | 0.96 (0.63, 1.46) | 0.87 (0.55, 1.37) | 0.85 (0.52, 1.38) | 0.94 (0.61, 1.45) | 0.083 | 0.043 |
| Medical, health care, welfare, education and public servant | 868 (21%) | 0.93 (0.67, 1.29) | 1.05 (0.72, 1.52) | 0.99 (0.65, 1.52) | 0.98 (0.71, 1.35) | 0.809 | 0.216 |
| Other services | 860 (20%) | 1.30 (0.94, 1.79) | 1.08 (0.74, 1.56) | 1.22 (0.81, 1.83) | 1.22 (0.89, 1.66) | 0.318 | 0.07 |
| **Educational attainments** |  |  |  |  |  |  |  |
| High school or below | 1094 (26%) | 1.00 (reference) | 1.00 (reference) | 1.00 (reference) | 1.00 (reference) | 0.334 | 0.044 |
| College, university or graduate school | 3128 (74%) | **0.72 (0.58, 0.90)** | 1.03 (0.81, 1.30) | 0.96 (0.73, 1.26) | **0.78 (0.63, 0.96)** | 0.334 | 0.044 |
| **Marital status** |  |  |  |  |  |  |  |
| Married | 2386 (57%) | 1.00 (reference) | 1.00 (reference) | 1.00 (reference) | 1.00 (reference) | 0.175 | 0.061 |
| Never married | 1542 (37%) | 1.13 (0.89, 1.45) | **0.69 (0.51, 0.92)** | 1.00 (0.72, 1.39) | 0.85 (0.67, 1.07) | 0.036 | 0.065 |
| Divorced or widowed | 294 (7%) | **1.45 (1.04, 2.01)** | **1.57 (1.05, 2.36)** | 1.28 (0.83, 1.97) | **1.76 (1.24, 2.48)** | 0.272 | 0.068 |
| **Housing** |  |  |  |  |  |  |  |
| Owns | 1433 (34%) | 1.00 (reference) | 1.00 (reference) | 1.00 (reference) | 1.00 (reference) | 0.056 | 0.046 |
| Does not own | 2789 (66%) | 0.88 (0.71, 1.10) | 1.02 (0.80, 1.31) | **1.21 (0.90, 1.62)** | 0.83 (0.67, 1.02) | 0.056 | 0.046 |
| **Self-rated health** |  |  |  |  |  |  |  |
| Good | 3795 (90%) | 1.00 (reference) | 1.00 (reference) | 1.00 (reference) | 1.00 (reference) | 0.289 | 0.04 |
| Poor | 427 (10%) | 0.82 (0.61, 1.09) | 1.31 (0.93, 1.84) | 0.98 (0.68, 1.43) | 1.05 (0.78, 1.41) | 0.289 | 0.04 |
| ^1^PR = Prevalence Ratio, CI = Confidence Interval^.^ | | | | | | | |
| *Maximum standardized pairwise difference, before and after inverse probability of treatment weighting. | | | | | | | |
